# Mesocortical dopamine system modulates mechanical nociceptive responses recorded in the rat prefrontal cortex

**DOI:** 10.1186/1471-2202-14-65

**Published:** 2013-07-02

**Authors:** Shoichi Sogabe, Yuki Yagasaki, Kitaro Onozawa, Yoriko Kawakami

**Affiliations:** 1 Department of Physiology, School of Medicine, Tokyo Women’s Medical University, 8-1 Kawada-cho, Shinjuku-ku, Tokyo 162-8666, Japan; 2 Department of Oral and Maxillofacial Surgery, Tokyo Women’s Medical University Medical Center East, 8-1 Kawada-cho, Shinjuku-ku, Tokyo 162-8666, Japan

**Keywords:** ACC, PFC, Dopamine, D_2_R, Pain, Parkinson disease, VTA

## Abstract

**Background:**

Psychological conditions affect pain responses in the human anterior cingulate cortex (ACC) according to brain imaging analysis. The rodent prefrontal cortex (PFC) including cingulate areas is also related to the affective dimension of pain. We previously reported PFC nociceptive responses inhibited by inputs from the amygdala, such as with dopamine (DA) D_2_ receptor (D_2_R) blockers, to show decreased effect on amygdala projections. In this study, we examined whether direct projections from the ventral tegmental area (VTA) to the PFC affect nociceptive responses in the PFC.

**Results:**

High frequency stimulation (HFS, 50 Hz, 30 s) delivered to the VTA produced long-lasting suppression (LLS) of nociceptive responses in the rat PFC including cingulate and prelimbic areas. Nociceptive responses evoked by mechanical pressure stimulation (2 s duration at 500 g constant force) applied to the tails of urethane-anesthetized rats were recorded using extracellular unit recording methods in the PFC. HFS delivered to the VTA, which has been reported to increase DA concentrations in the PFC, significantly suppressed nociceptive responses. The LLS of nociceptive responses persisted for about 30 minutes and recovered to the control level within 60 min after HFS. We also demonstrated local microinjection of a selective D_2_ agonist of DA receptors to induce LLS of mechanical nociceptive responses, while a D_2_ but not a D_1_ antagonist impaired the LLS evoked by HFS. In contrast, DA depletion by a 6-hydroxydopamine injection or a low concentration of DA induced by a κ-opiate receptor agonist injected into the VTA had minimal effect on nociceptive responses in the PFC.

**Conclusion:**

HFS delivered to VTA inhibited nociceptive responses for a long period in PFC. DA D_2_R activation mediated by local D_2_ agonist injection also induced LLS of mechanical nociceptive responses. The mesocortical DA system may modify PFC nociceptive responses via D_2_ activity.

## Background

Psychological conditions like attention [1] and hypnotic effects [2] exert powerful influences on human pain sensations. The anterior cingulate cortex (ACC) has crucial roles in conscious perception of pain but not the sensory discriminative aspect of pain [3]. Noxious stimulation applied to peripheral tissues evoked nociceptive responses in rabbit and rodent cingulate and prelimbic areas [4,5], lesions of which impaired place avoidance test responses while pain behavior on the formalin test was normal [6]. The human ACC and the rodent prefrontal cortex (PFC) receiving inputs from the amygdala are involved in emotional processing [7]. In our previous study [8], nociceptive responses of the PFC were inhibited by inputs from the amygdala. We also found that a dopamine (DA) D_2_ receptor (D_2_R) blocker impaired the inhibition induced by amygdala stimulation. Direct dopaminergic projections from the ventral tegmental area (VTA) to the PFC are responsible for modulatory effects on nociceptive responses [9], suggesting that the mesocortical DA system modulates pain responses in the PFC.

Recent studies have explored how DA modulates pain perception [10]. Systemic administration of DA antagonists were reported to alter nociceptive responses [11]. Symptoms of pain in Parkinson disease (PD) imply that DA levels in the brain affect pain sensations. In clinical reports on PD, patients frequently complain of neuropathic or central pain before motor disorders [12-14]. These clinical reports indicate that the brain DA level modifies pain sensations. In animal studies, microinjections of DA into the ACC reduced autotomy scores in a sciatic neurotomy model [15]. Behavioral in vivo studies of pain have demonstrated that DA depletion induces significant changes in thresholds for noxious stimuli [16,17]. Six-hydroxydopamine (6-OHDA) lesions in the VTA induced hyperalgesia in acute and chronic pain models [18]. We determined whether lower DA concentration changes in the VTA induced by 6-OHDA lesions alter nociceptive responses in the PFC. We also examined effects of a κ-opiate receptor agonist injected into VTA on nociceptive responses. Dopaminergic projections from VTA have two main target areas, PFC and the nucleus accumbens. A κ-opiate receptor agonist injected into VTA inhibited only DA neurons projecting to PFC and reduced DA level in PFC [19].

The rodent PFC receives DA projections from the VTA. The present study demonstrated VTA-PFC projections to directly affect nociceptive responses recorded in the PFC. VTA projections terminated at synapses within the soma, dendritic shafts, and spines of pyramidal cells in layers II to V of the PFC [20,21], while nociceptive information was transmitted through the medial pain pathways from the periphery to the PFC [5,22]. The crucial roles of DA in PFC function were discussed in the review by Seamans [23]. The mesocortical DA pathways from the VTA have modifying effects on cognition [24] in the PFC. The VTA-PFC pathways may affect activities of the medial pain pathways, of which the PFC is the center. We analyzed the effects of dopaminergic inputs from the VTA to the prelimbic and cingulate areas on nociceptive responses in the PFC. We also clarified which of the DA receptor subtypes is related to the modulatory effects on nociceptive responses in the PFC.

## Results

### Mechanical noxious stimulation induced nociceptive responses in the PFC

**Figure 1:**
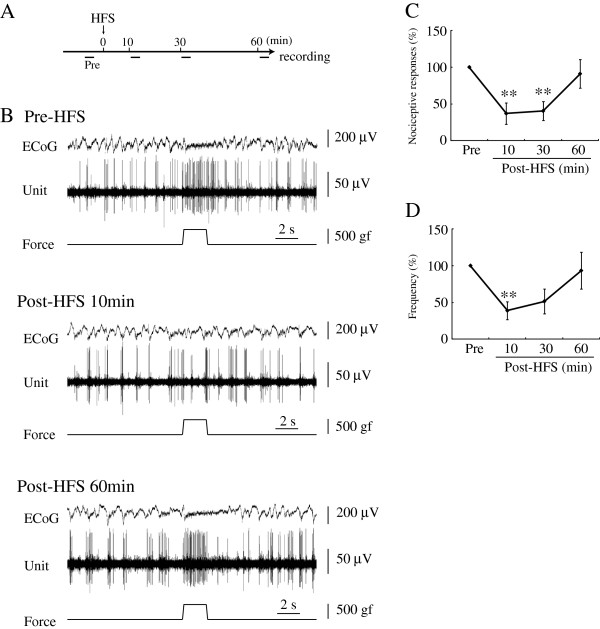
**HFS delivered to the VTA suppressed nociceptive responses recorded in the PFC. A:** Time course of procedure is presented. Pre: Pre-HFS. **B:** The top trace represents ECoG. The second trace is multiple unit discharges. The bottom trace represents a pressure curve. Nociceptive responses were depressed by HFS delivered to the VTA at 10min. At 60 min after HFS, nociceptive responses recovered to the pre-HFS control level. **C, D:** Inhibitory effects of VTA on nociceptive responses induced by HFS delivered to the VTA. Mean changes in the duration (C) and frequency (D) of responses are presented. **p<0.01, Error bars represent S.E. n=11.

Mechanical noxious stimulation induced excitatory responses in PFC neurons, which persisted during and frequently after stimulation. Two types of nociceptive responses, a wide dynamic range type and a specific high threshold (SHT) type, were recorded [5]. In this study, we used SHT neurons to monitor the response to noxious stimulation. The spontaneous background discharges of neurons, which usually showed the spindle bursts characteristic urethane anesthesia, continued for more than two hours, if the anesthetic level was maintained like we had previously reported [5,8]. We administered additional urethane when typical spindle bursts disappeared. Electrocorticography (ECoG) also showed typical high amplitude slow waves and was changed to low amplitude fast waves by mechanical stimulation (Figure 1B). Anesthetic levels were also detected with ECoG patterns. The nociceptive responses were recorded without adaptation, if mechanical stimulation was applied every 90 s.

### HFS delivered to the VTA decreased nociceptive responses in the PFC

High frequency stimulation (HFS, 50 Hz, 30 s) delivered to the VTA impaired PFC nociceptive responses evoked by mechanical stimulation applied to the rat tail (11 units/9 rats) (Figure 1B). Durations of nociceptive responses were 3.00 ± 0.46 s pre-HFS, and 0.82 ± 0.33 s (36.9 ± 14.5% of pre-HFS) at 10 min (p<0.01), 1.05 ± 0.29 s (40.4 ± 12.7%) at 30 min (p<0.01) and 2.55 ± 0.60 s (91.0 ± 19.3%) at 60 min post-HFS (Figure 1C). Frequencies of discharge were 5.43 ± 1.00 pre-HFS, and 2.06 ± 0.78 (38.8 ± 12.3% of pre-HFS) at 10 min (p<0.01), 2.71 ± 1.01 (51.4 ± 16.8%) at 30 min and 4.34 ± 1.03 (93.3 ± 25.1%) at 60 min post-HFS (Figure 1D). Long-lasting suppression (LLS) of nociceptive responses appeared within 10 min and persisted for 30 min after HFS. Nociceptive responses recovered to pre-HFS levels in 60 min. HFS delivered to the VTA clearly inhibited nociceptive responses in the rat PFC.

### D_2_R antagonist blocked the depression of nociceptive responses

**Figure 2:**
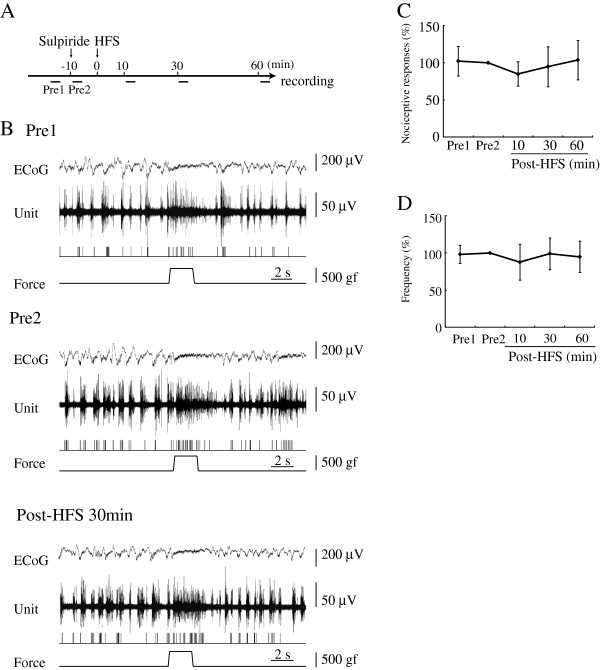
**Microinjection of a D**_**2**_**R antagonist modified the depression of nociceptive responses induced by HFS. A:** Time course of procedure is presented. Pre1: Pre-HFS (before sulpiride microinjection). Pre2: Pre-HFS (after sulpiride microinjection). **B:** The top trace represents ECoG. The second trace is multiple unit discharges evoked by mechanical stimulation. The third trace represents single unit responses selected by cluster analysis from multiple units in the top line. The bottom trace represents a pressure curve. The D_2_R antagonist, sulpiride, impaired effects of HFS delivered to the VTA. **C, D:** At 10 and 30 min after HFS, a D_2_R antagonist blocked the depression of nociceptive responses induced by HFS to the VTA to a statistically significant extent. Mean changes in the duration **(C)** and frequency **(D)** of responses are presented. Error bars represent S.E. n=9.

A D_2_R antagonist, sulpiride, which had no effect on control nociceptive responses, significantly blocked the inhibitory effects of HFS (9 units/8 rats). Durations of pain responses were 2.43 ± 0.46 s pre-microinjection and 2.69 ± 0.67 s post-microinjection. There was no significant difference in nociceptive responses or background ECoG between the two phases (pre & post D_2_R antagonist injection) (Figure 2B). After HFS had been delivered to the VTA, the durations of nociceptive responses were 2.28 ± 0.45 s (85.0 ± 16.4% of pre-HFS) at 10 min, 2.36 ± 0.62 s (94.8 ± 26.8%) at 30 min and 2.56 ± 0.41 s (103.7 ± 26.6%) at 60 min post-HFS (Figure 2C). Frequencies of discharge were 4.37 ± 1.00 s pre-HFS, post-microinjection, and 4.54 ± 1.64 (87.8 ± 24.1% of pre-HFS) at 10 min, 4.64 ± 1.30 (99.1 ± 21.4%) at 30 min and 4.43 ± 1.50 (95.0 ± 21.1%) at 60 min post-HFS (Figure 2D). In the sulpiride-treated group, the durations and frequencies of pain responses showed no statistically significant differences.

### D_1_R antagonist had no effect on nociceptive responses in the PFC

**Figure 3:**
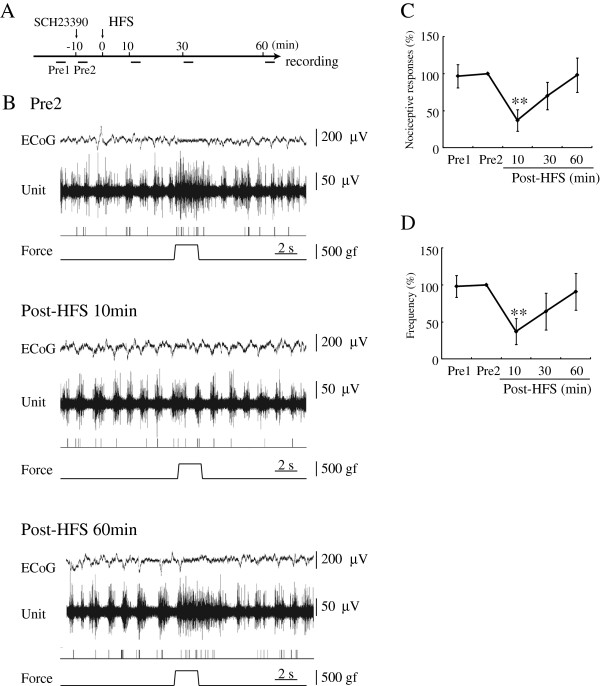
**Microinjection of D**_**1**_**R antagonist had no effect on the inhibitory effects of HFS. A:** Time course of procedure is presented. Pre1: Pre-HFS (before SCH23390 microinjection). Pre2: Pre-HFS (after SCH23390 microinjection). **B:** The top trace represents ECoG. The second trace is multiple unit discharges evoked by mechanical stimulation. The third trace represents single unit responses selected by cluster analysis from multi-units of the top trace. The bottom trace represents a pressure curve. Nociceptive responses were depressed by HFS delivered to the VTA at 10min. At 60 min after HFS, nociceptive responses showed recovery to the pre-HFS control level. **C, D:** Mean changes in the duration **(C)** and frequency **(D)** of responses are presented. **p<0.01, Error bars represent S.E. n=8.

A D_1_ receptor (D_1_R) antagonist, SCH23390, had no effect on the inhibitory effects of HFS (8 units/6 rats). Durations of pain responses were 1.94 ± 0.43 s pre-microinjection and 1.97 ± 0.25 s post-microinjection. There was no significant difference in nociceptive responses or background ECoG between the two phases (pre & post D_1_R antagonist injection) (Figure 3B). After HFS had been delivered to the VTA, the durations of nociceptive responses were 0.75 ± 0.31 s (37.0 ± 14.6% of pre-HFS) at 10 min (p<0.01), 1.38 ± 0.35 s (70.0 ± 18.4%) at 30 min and 2.13 ± 0.70 s (98.3 ± 23.2%) at 60 min post-HFS (Figure 3C). Frequencies of discharge were 3.49 ± 0.57 pre-HFS, post-microinjection, and 0.88 ± 0.30 (37.1 ± 17.8% of pre-HFS) at 10 min (p<0.01), 1.78 ± 0.59 (64.2 ± 24.8%) at 30 min and 2.66 ± 0.61 (90.9 ± 25.0%) at 60 min post-HFS (Figure 3D).

### D_2_R agonist decreased nociceptive responses in PFC

**Figure 4:**
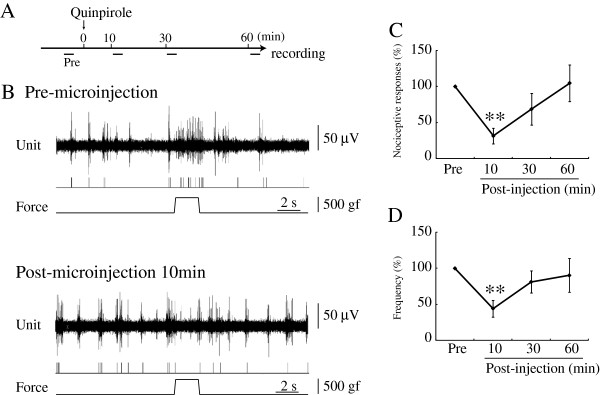
**Microinjection of D**_**2**_**R agonist suppressed nociceptive responses recorded in the PFC. A:** Time course of procedure is presented. Pre: Pre-quinpirole microinjection. **B:** The top trace is multiple unit discharges evoked by mechanical stimulation. The second trace represents single unit responses selected by cluster analysis from multi-units. The bottom trace represents a pressure curve. Nociceptive responses were depressed by D_2_R agonist, quinpirole, microinjection at 10 min. **C, D:** Mean changes in the duration **(C)** and frequency **(D)** of responses are presented. **p<0.01, Error bars represent S.E. n=9.

A D_2_R agonist, quinpirole, decreased nociceptive responses in the PFC (9 units/5 rats) (Figure 4B). Durations of pain responses were 1.54 ± 0.20 s pre-microinjection, and 0.44 ± 0.13 s (31.4 ± 10.9% of pre-HFS) at 10 min (p<0.01), 0.94 ± 0.28 s (68.6 ± 22.0%) at 30 min and 1.44 ± 0.24 s (104.8 ± 25.4%) at 60 min post-microinjection (Figure 4C). Frequencies of discharge were 6.09 ± 1.31 pre-microinjection, and 2.43 ± 0.72 (44.0 ± 11.5% of pre-HFS) at 10 min (p<0.01), 4.16 ± 0.94 (81.1 ± 15.4%) at 30 min and 3.67 ± 0.97 (90.2 ± 23.4%) at 60 min post-microinjection (Figure 4D). LLS of nociceptive responses appeared within 10 min after microinjection. Nociceptive responses recovered to pre-microinjection levels in 60 min. Microinjection of a D_2_R agonist clearly inhibited nociceptive responses in the PFC.

### κ-opiate receptor agonist microinjection into the VTA

**Figure 5:**
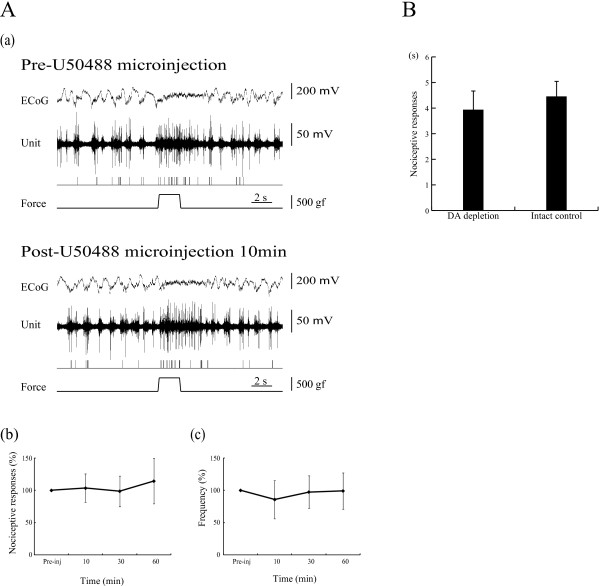
**Microinjection of κ-opiate receptor agonist and DA depletion had no effect on nociceptive responses in PFC. A: (a)** The top trace represents ECoG. The second trace is multiple unit discharges evoked by mechanical stimulation. The third trace represents single unit responses selected by cluster analysis from multi-units. The bottom trace represents a pressure curve. Mean changes in the duration **(b)** and frequency **(c)** of responses are presented. Error bars represent S.E. n=9. **B:** Nociceptive discharges were recorded in the PFC of the side ipsilateral to 6-OHDA injection. There was no difference in mean nociceptive responses between the control (n= 8) and 6-OHDA groups (n=10). Error bars represent S.E.

A κ-opiate receptor agonist, U50488, microinjected into the VTA had no effect on nociceptive responses or background ECoG in the PFC (9 units/7 rats) (Figure 5A(a)). Durations of pain responses were 1.94 ± 0.44 s pre-microinjection, and 1.67 ± 0.46 s (103.5 ± 22.0% of pre-microinjection) at 10 min, 1.83 ± 0.51 s (98.7 ± 23.7%) at 30 min and 1.89 ± 0.48 s (114.4 ± 38.4%) at 60 min post-microinjection (Figure 5A(b)). Frequencies of discharge were 3.99 ± 0.43 pre-microinjection, and 3.84 ± 1.63 (85.7 ± 29.7% of pre-microinjection) at 10 min, 3.71 ± 0.77 (97.4 ± 25.2%) at 30 min and 4.32 ± 1.35 (99.1 ± 28.3%) at 60 min post-microinjection (Figure 5A(c)).

### DA depletion had no effect on nociceptive responses in the PFC

DA depletion was established by apomorphine tests three weeks after 6-OHDA injection into the medial forebrain bundle (MFB). In apomorphine test-positive animals, background ECoG showed low voltage fast waves, as described in a previous report [25]. However, nociceptive responses were normally evoked by mechanical stimulation delivered to the tail. There was no difference in mean nociceptive responses between the control (n=8) and 6-OHDA groups (n=10) (4.51 ± 0.49 s and 3.93 ± 0.68 s, respectively) (Figure 5B).

## Discussion

Our study examined whether the mesocortical DA system directly affects nociceptive responses in the PFC. HFS delivered to the VTA inhibited nociceptive responses recorded in the PFC. Basically, the DA concentration in the PFC, which receives direct inputs from the VTA [20,21], is tonically maintained by activities of dopaminergic neurons in the VTA. HFS delivered to the VTA [26] and activation of VTA neurons by N-methyl-D-aspartate (NMDA) [27] increase the extracellular DA concentration, thereby producing inhibitory effects on PFC neurons [28]. Higher DA concentrations induced by HFS may produce inhibition of nociceptive responses in the PFC. The inhibition, which was induced by a HFS of a moderate intensity, persisted for 60 min. In PFC slice studies, bath application of DA strongly modulated long-term depression of glutamatergic synapses [29]. Our previous in vivo study [8] indicated that DA modulated LLS induced by glutamate receptor activation. Burst stimulation of VTA increased DA release in PFC, which induced plasticity of PFC neurons [30]. DA release by HFS delivered to VTA may induce plastic changes in glutamatergic synapses to receive nociceptive inputs from peripheral tissue.

In contrast, DA depletion induced by 6-OHDA [31] or a decrease in the DA concentration induced by κ-opiate application [19] had no effect on nociceptive responses in the PFC. DA depletion by 6-OHDA increased the low amplitude and high frequency components in background ECoG, as reported previously [25]. Decreased tonic DA release suppressed D_2_-mediated activities followed by augmentation of PFC neuron activities [32]. These reports indicate that a low level of DA affects spontaneous activities of PFC neurons, but effects on evoked responses remain unclear. Our results and those of a previous report [8] demonstrated a low DA concentration in the PFC to impair plastic changes but not normal nociceptive responses.

In the rodent PFC, local application of DA reportedly induced spike activities, while a D_2_R antagonist impaired suppression of spontaneous discharges [28,33,34]. Our results from HFS to the VTA and local injection of a D_2_R agonist indicate D_2_R activation to be responsible for the LLS of nociceptive responses induced by HFS of the VTA. A D_1_R antagonist, however, had minimal effects on nociceptive responses, as reported by Godbout [28]. DA D_1_R and D_2_R are found on both pre- and post-synaptic pyramidal neurons in the PFC [35-37]. D_1_R activating adenylate cyclase through interactions with G-proteins (Gs) [38] enhanced the NMDA current [39], which induced long-term potentiation (LTP) in the PFC [40]. A question is why HFS did not evoke excitatory effects via D_1_Rs activities. More D_1_Rs are expressed on gamma-aminobutyric acid (GABA) neurons than on pyramidal cells in the PFC [41], suggesting that D_1_R activities evoked by HFS did not consistently induce excitatory responses. Another possibility is that DA D_1_R produces an inverted-U shaped response, indicating a higher dose to be associated with somewhat lower performance [42,43]. The DA concentrations induced by HFS of the VTA may evoke DA D_2_- but not DA D_1_-mediated activities in PFC neurons.

The PFC receives numerous projections from several areas involved in complex higher brain functions. The medial pain pathways extend from the periphery to the PFC [5,22] and direct projections from the VTA terminate in the PFC. Both inputs converged in the same areas (Additional file 1) where unit discharges were recorded in this study. Projections from the amygdala and hippocampus also terminate in the PFC and thereby change pain responses [8,44]. These areas are related to emotion and memory, suggesting that the PFC unifies affectional information and pain. According to human brain imaging analyses, the strength of conscious pain, which is related to psychological condition, reflects activities of the PFC [45]. DA modified synaptic plasticity [8,46] and prolonged DA-mediated modulation biases the long-term processing dynamics of PFC networks [30].

## Conclusion

Mechanical noxious stimulation applied peripherally evoked nociceptive discharges in the PFC. HFS delivered to the VTA inhibited persistently nociceptive responses recorded in the PFC. This long lasting inhibition was decreased by a DA D_2_R antagonist. In addition, a D_2_R agonist alone produced inhibition of nociceptive responses. The DA system (VTA-PFC projections) exerts modulatory effects on pain responses recorded in the PFC. The mesocortical DA system may affect complex pain cognition.

## Materials and methods

### Animal preparation

Adult male Wistar rats (270-350 g; Sankyo Laboratory Co., Tokyo, Japan) were used in all experiments. The rats were housed under controlled temperature (25°C) and humidity (40-50%) conditions with a 12-h light/dark cycle, and had free access to food and water. Experiments conformed to guidelines issued by the National Institutes of Health for Laboratory Animals and all procedures were approved by the Animal Experimental Committee of Tokyo Women’s Medical University. Efforts were made to minimize the number of animals used and their suffering. All rats were anesthetized with a single injection of urethane (1.5 g/kg, i.p.) and mounted in a stereotaxic instrument (Narishige, Tokyo, Japan) for the acute experiments.

### Mechanical stimulation

Mechanical pressure was applied to the tail (1.0-2.0 cm distal to the body) employing a mechanical stimulator (DPS-270; DIA Medical System Co., Tokyo, Japan), using a probe with a circular contact area with a 1 mm-in-diameter tip. Mechanical stimuli were delivered every 90 s at constant force with a feedback system. Stimulus intensity in this experiment was 500 gf with a 0.1 s rising (and decreasing) time to maximum force and a 2 s hold time. The nociceptive stimulus intensity of 300 gf has been shown to be adequate [47] to induce C-fiber mediated activity in peripheral nerves [48]. These mechanical stimulus conditions induced stabile nociceptive responses lasting more than two hours under the same anesthetic levels. (For details, refer to our previous reports [5,8]).

### Electrophysiological recording

**Figure 6:**
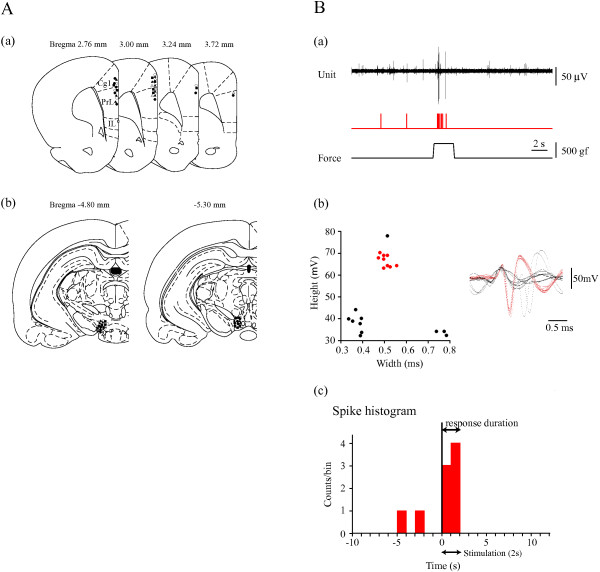
**Recording site and stimulus point locations and data analysis. A: (a)** Recording sites in the PFC. All unit discharges are scattered throughout the cingulate and prelimbic areas. Solid circles represent the recording sites. Drug injection and recording sites (solid triangles: D_2_R antagonist, solid squares: D_1_R antagonist, and open triangles: D_2_R agonist). The numbers represent the distance from the Bregma. Cg1: cingulate cortex area 1. PrL: prelimbic cortex. IL: infralimbic cortex. **(b)** Stimulus and drug injection sites in the VTA. Solid circles represent the locations to which HFS was delivered. Open circles represent the locations at which the κ-opiate receptor agonist was injected. **B: (a)** The top trace is multiple unit discharges. The second trace represents discriminated single units. The bottom trace represents a pressure curve. Nociceptive stimulation activated discharges. **(b)** Cluster analysis of single units (red) with wave width and height on the left. Right side traces represent waves on the left figure. Red units on the left have the same forms. **(c)** Responses are shown as a histogram with 1sec bin after single units were discriminated. Vertical lines represent the stimulus starting point.

Recording tungsten needle microelectrodes (impedance 8-12 MΩ; FHC, ME, USA) were stereotaxically positioned in the cingulate or prelimbic areas of the PFC. Stereotactic coordinates of the PFC were 2.8-4.2 mm anterior and 0.3-0.8 mm lateral to the Bregma [49]. The perpendicular depths of the recording sites were between 0.2 and 2.2 mm from the dorsal cortical surface (Figure 6A(a)). The unit spikes were processed with a multichannel amplifier (MEG-6100; Nihon Kohden Co., Tokyo, Japan; 0.08-3000 Hz) and an active filter (DV-04; NF Electronic Instruments Co., Yokohama, Japan; 500-3000 Hz), respectively. Through a memory oscilloscope (VC-11; Nihon Kohden Co.), the data were fed into a thermal array recorder (RTA-1100M; Nihon Kohden Co.) for paper recording and a personal computer (Vostro420; Dell, TX, USA) via an integrated system (PowerLab/4SP; Mountain View, CA, USA) for recording storage and later off-line analysis. The spontaneous background discharge patterns and ECoG were recorded through the same electrodes. Anesthetic levels and nociceptive responses were determined with ECoG. We administered additional urethane when typical spindle bursts disappeared. ECoG changed from slow waves with spindle bursts to low amplitude fast waves induced by mechanical stimulation (Figure 1B).

### Local application of drugs

A cannula (0.18 mm inside diameter, Teflon) with a stainless recording electrode (TF205-074, Unique Medical Co., Tokyo, Japan) was stereotaxically positioned in the left PFC or VTA. The cannula was connected by polyethylene tubing (30 cm length) to a 25 μl syringe (Hamilton, NV, USA). The drug was microinjected by means of a syringe pump at a rate of 1 μl/min (55-1111; Harvard Apparatus Co., MA, USA). All drugs were dissolved in 0.9% saline. The following barrel concentrations were used: 5 μM/5 μl sulpiride (D_2_R antagonist; Sigma, St. Louis, MO, USA), 5 μM/5 μl quinpirole (D_2_R agonist; Sigma), 100 nM/6 μl SCH23390 (D_1_R antagonist; Sigma), 100 μM/5 μl U-50488 (κ-opiate receptor agonist; Sigma). Sulpiride, SCH23390 and quinpirole were locally injected into the PFC. A κ-opiate receptor agonist, U-50488, was injected into the VTA to decrease DA concentrations [19].

### HFS applied to the VTA

In these experiments, tungsten needle microelectrodes (impedance 8-12 MΩ; FHC) were positioned in the VTA. Stereotactic coordinates of the VTA were 4.5-5.5 mm posterior and 0.3-0.8 mm lateral to the Bregma. The perpendicular depths of the recording sites were between 7.5 and 8.5 mm from the dorsal cortical surface (Figure 6A(b)). Electrical HFS (50 Hz, 250 μA, 100μs square pluses, for 30 s) was applied to the VTA to increase DA concentrations in the PFC [50].

### Depletion of DA

Rats were anesthetized with Nembutal (50 mg/kg i.p.). Then, five μl of 6-OHDA HCl (2 mg in 1ml of saline containing 0.1% ascorbic acid; Sigma) were injected into the left MFB through a cannula with a microinjection pump at a rate of 20 μl/hr, and the cannula was left in place for 10 min after the completion of pumping. Stereotactic coordinates of the MFB were 4.5 mm posterior and 1.1 mm lateral to the Bregma. The perpendicular depths of the recording sites were 8.2 mm from the dorsal cortical surface. At the end of injection, the cannula was removed and the skull skin was sutured. We assessed motor disturbance 3 weeks after 6-OHDA injection by circling behavior (LE 902/Rp Container; Panlabs.I., Barcelona, Spain) with apomorphine (1 mg/kg i.p.) administration. The same acute experimental procedures as described above (Recording and stimulating electrodes) were carried out after DA depletion had been established. Nociceptive discharges were recorded in the PFC of the side ipsilateral to 6-OHDA injection. Apomorphine induced rotational asymmetry was not observed in the intact group (Pre-6-OHDA treatment) serving as the control. Before the 6-OHDA injections, we performed apomorphine tests (1 mg/kg i.p.) to avoid spontaneous DA abnormalities.

### Unit recording locations

The locations of units were marked with a positive electric current lesion (direct current, 100 μA for 15 s). At the end of each experiment, the animals were perfused with normal saline and 4% paraformaldehyde. After overnight post-fixation, the brains were sectioned (50 μm) and stained with Cresyl Violet solution to examine the recording sites under light microscopy.

### Data analysis

A single unit spike was discriminated on the basis of the height and width of each unit from a multi-unit recording obtained (scatter plotting Figure 6B(b) left) and the same single units were determined from clusterized discharges with pattern matching (Figure 6B(b) right). Software LabChart 7.0 (AD Instruments Co., Tokyo, Japan) was used for unit discharge analysis. The spike histogram was analyzed using single spikes during a 22s period (10s before stimulation and 10s after stimulation, Figure 6B(c)). Each bin of histograms consists of spikes during a 1 s period. The durations of responses exceeding double the mean spontaneous discharges on the histogram were assessed as the response durations (Figure 6B(c)). Significant differences in discharges evoked by mechanical stimuli were assessed with the nonparametric paired-test (Wilcoxon) to compare pre- and post- stimulation values. Data are expressed as means ± standard errors (S.E.). A probability level of <0.05 was considered significant.

## Abbreviations

ACC: Anterior cingulate cortex; Cg1: Cingulate cortex area 1; D1R: D_1_ receptor; D2R: D_2_ receptor; DA: Dopamine; ECoG: Electrocorticography; GABA: Gamma-aminobutyric acid; HFS: High frequency stimulation; IL: Infralimbic cortex; LLS: Long-lasting suppression; LTP: Long-term potentiation; MFB: Medial forebrain bundle; NMDA: N-methyl-D-aspartate; 6-OHDA: 6-hydroxydopamine; PD: Parkinson disease, PFC, prefrontal cortex; Prl: Prelimbic cortex; SHT: Specific high threshold; VTA: Ventral tegmental area.

## Competing interests

None of the authors have any conflicts of interest to report.

## Authors’ contributions

SS, YY and KO carried out the experiments. SS, YY and KO analyzed the experimental data. SS wrote the manuscript. YK conceived the study, designed the experiments and helped to draft the manuscript. All authors have read and approved the final manuscript.

## Supplementary Material

Additional file 1 Convergence of VTA and medial pain pathways in the superficial layer of the PFC. Click here for file
