# Supplementary material for: Mesocortical dopamine system modulates mechanical nociceptive responses recorded in the rat prefrontal cortex
Source: BMC Neurosci. 2013 Jul 2;14:65. doi: 10.1186/1471-2202-14-65 (PMC3710228; doi:10.1186/1471-2202-14-65)
Supplement: Additional file 1 — Convergence of VTA and medial pain pathways in the superficial layer of the PFC. [file 1471-2202-14-65-S1.pdf]

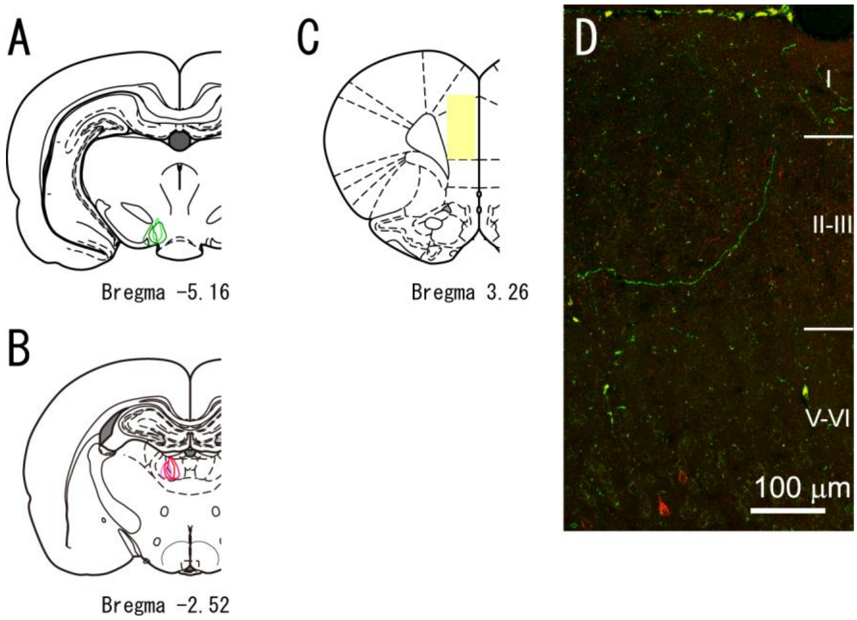

Supplemental Figure 1: Convergence of VTA and medial pain pathways in the superficial layer of the PFC

Injected Neuro Vue Maroon was largely restricted to the VTA (Fig. A) and only cases with Neuro Vue Red injections centered in the mediodorsal thalamic nucleus (MD) and centrolateral thalamic nucleus (CL) (Fig. B) were examined in this study. The examination area of PFC was indicated in Fig. C. Injection of Neuro Vue Maroon into the VTA resulted in anterograde labeling of fibers in all layers of the PFC and retrograde labeling of cells in layers V-VI of the PFC (Fig. D, green). Injection of Neuro Vue Red into the MD and CL resulted in anterograde labeling of fibers mainly in layers I-III of the PFC and retrograde labeling of cells in layers V-VI ((Fig. D, red). Both the terminal field of Neuro Vue Maroon-labeled VTA fibers and Neuro Vue Red-labeled MD and CL fibers were seen in layers II-III of the PFC (Fig. D).

Supplemental Method

Neuronal tract tracing

Rats were anesthetized with Nembutal (50 mg/kg i.p.). Then, a unilateral cannula (0.18 mm inside diameter, Teflon) with a recording electrode (FHC, ME, USA) was stereotaxically positioned in the left VTA and thalamus (MD and CL). The cannula was connected by polyethylene tubing (30 cm length) to a 25 μl syringe (Hamilton, NV, USA). Stereotactic coordinates of the thalamus (MD and CL) were 2.6 mm posterior and 0.8-1.2 mm lateral to the Bregma. The perpendicular depths of the recording sites were between 5.4 and 5.6 mm from the dorsal cortical surface. 100 nl of Neuro Vue Maroon (Polyscience, PA, USA) were injected into the left VTA. Similarly, 100 nl of Neuro Vue Red (Polyscience) were injected into the left thalamus. The drugs were microinjected at a rate of 0.8 μl/hr (55-1111; Harvard Apparatus CO., MA, USA), and the cannulas were left in place for 20 min after the completion of pumping. At the end of injection, the cannulas were slowly removed and the skin was sutured. Two weeks after injection, the rats were deeply anesthetized with Nembutal (50 mg/kg i.p.) and intra-cardially perfusion-fixed with freshly prepared 4% paraformaldehyde in 0.1 M phosphate buffer. After perfusion, the brains were post-fixed in 4% paraformaldehyde overnight. These brains were sectioned with a vibrating blade microtome (VT 1000S, Leica, Germany) at a 50 μm (PFC projection site) or 100 μm (each injection site) thickness. Confocal images of the PFC projection site were taken using LSM 510 with LSM Image Browser version 3.5 software (Carl Zeiss, Germany). The fluorescent micrographic images and light micrographic images of each injection site were taken using a BX-57 digital microscope with DP controller software (Olympus, Tokyo, Japan). Data were obtained from three rats receiving appropriate tracer injections.
